# The place of family medicine and primary health care in South Sudan’s fragile health system

**DOI:** 10.4102/phcfm.v17i1.5157

**Published:** 2025-10-08

**Authors:** Machuor D. Arok, Kenneth L. Sube, Susan P. Lado

**Affiliations:** 1 Department of Community Medicine, School of Medicine, University of Juba, Juba, South Sudan; 2 Division of Family Medicine and Primary Health Care, Faculty of Medical Services, Family Doctors Africa, Juba, South Sudan; 3 School of Medicine, University of Juba, Juba, South Sudan

**Keywords:** family medicine, primary health care, health system, fragile, South Sudan

## Abstract

Since independence in 2011, South Sudan has faced recurrent conflict, economic collapse and a protracted humanitarian crisis. The health system is fragile, underfunded and donor dependent, with only 40% – 45% of the population accessing functioning facilities. Primary health care (PHC) forms the foundation of service delivery but is constrained by poor infrastructure, shortages of skilled workers and a narrow service scope. Medical education is similarly limited, with few specialist training opportunities and a critical shortage of family physicians. This review synthesises national health system data, literature on family medicine (FM) in sub-Saharan Africa and contextual analysis of South Sudan’s PHC and medical education landscape to explore opportunities for integrating FM into the national system. Evidence from other African countries shows FM improves access to continuous, person-centred care; strengthens integration; and builds resilience during crises. In South Sudan, FM could address high maternal and child mortality, expand community-based care, and enhance workforce capacity. Opportunities include: (1) the large share of healthcare needs met at primary level, (2) existing undergraduate Community Medicine programmes as a foundation for FM training, and (3) the establishment of the South Sudan Association of Family Physicians (SSAFP) to promote advocacy, postgraduate training and regional collaboration. Integrating FM into South Sudan’s PHC system offers a strategic pathway towards equitable, sustainable and resilient healthcare. Building on existing structures, fostering partnerships and investing in postgraduate FM education could accelerate progress towards universal health coverage and long-term health system recovery in a post-conflict context.

## Background

After gaining her independence from Sudan in 2011 following five decades of civil war, South Sudan faced internal conflict in 2013. This led to another devastating civil war. The 2018 Revitalized Peace Agreement on the Resolution of Conflict in South Sudan brought some stability, although challenges remain in implementing peace.^1^ The country is governed by a transitional power-sharing government, with political tensions, ethnic divisions and weak institutions hindering progress.

Oil production accounts for about 90% of government revenue.^2^ However, fluctuating oil prices and production disruptions have hampered economic growth. Hyperinflation and a depreciating currency have severely impacted citizens’ purchasing power, with over 80% of the population living below the poverty line.^3^ Most people depend on subsistence farming, livestock and fishing, but poor infrastructure and insecurity hinder the transportation of commodities to the market and thus restrict productivity. With a population of 13.4 million,^4^ over 70% under 30 years of age,^5^ South Sudan has a youthful population with a brighter future that can impact change, development and innovations. However, the country experiences one of the world’s worst humanitarian crises. Millions are internally displaced with women and children disproportionately suffering violence, poverty and gender-based violence.^3^ The majority of the population relies on humanitarian aid and international support for food, healthcare and education.

## South Sudan’s health system

The country’s healthcare system is particularly fragile. This is because of high maternal and infant mortality, low vaccination coverage, inadequate access to crucial health services, poor infrastructure, out-of-pocket usage accounting for 54% of the annual health budget, limited financial resources, inadequate skilled health workers and improvised governance. Moreover, the government funding for health is below the Abuja declaration (less than 2% of the annual national budget in comparison to 15% per the Abuja declaration).^6^ Educational opportunities are also limited, particularly in rural areas. According to the 2024 intersectoral needs analysis, only 40% of the studied population can access the nearest health facility by walking for one hour, while 45% face long waiting times as a barrier to care. However, 63% consider the inability to pay for health services to be a barrier accessing health care.^7^

Women and girls in remote and crisis-affected areas struggle to access maternal, sexual and reproductive health services.^8^ The Health Sector Transformation Programme, a donor-funded programme supports 50% of functional health facilities, but 18% remain non-functional or closed down because of a lack of funding.^3^ Fifty-nine per cent of the population lacks access to safe water, and only 10% has access to improved sanitation. Only 7.2% of the population has access to electricity.^9^

## Primary health care in South Sudan

South Sudan has structured its health system around the World Health Organization’s primary health care (PHC) recommendation, with services organised from community to national levels: Boma Health Services led by community health workers, primary health care units (PHCUs) at the Boma (smallest administrative unit, same as village) level serving 2000–10 000 people and run by registered nurses, primary health care centres (PHCCs) at the Payam (sub-county) level for 10 000–30 000 people staffed by clinical officers and County Hospitals covering 80 000–200 000 people led by medical officers. At the secondary level, state hospitals serve 300 000–1 000 000 people, while National Referral Hospitals provide specialised care for the entire country.^10^ This arrangement is intended to provide basic promotive, preventive and curative services with PHCUs as the initial point of contact and PHCCs offer a more comprehensives health care services. The Boma Health Initiative, as a community base initiative, focuses on community participation and strengthening community health structures.

Despite this structure, lower-level facilities struggle with inadequate staffing, weak infrastructure and poor transport networks, which limit early intervention and make referrals difficult, worsening health outcomes. The weaknesses and challenges of South Sudan’s health system are summarised in Table 1. Healthcare is significantly underfunded, with only 3% of the 2024/2025 Annual National Budget allocation.^11^ Despite the underfunding, the country continues to battle multiple occasional disease outbreaks, including measles, yellow fever, poliovirus, meningitis, hepatitis E and cholera. Malaria remains the leading cause of illness and death, worsened by frequent flooding. The maternal death rate is one of the highest in the world, with 1223 maternal deaths per 100 000 live births. Only 25% of women give birth at a health facility or with a skilled birth attendant. More than one and a half million children are acutely malnourished, and 10% of the children die before their 5th birthday.^9^

**TABLE 1:** South Sudan’s health system challenges.

Domain	Challenge(s)
Coverage and access	Only ~40% – 45% of the population have *physical access* to functioning health services; many communities live > 5 km from any facility; flooding and conflict cut off others seasonally.
Infrastructure	Many PHCUs and PHCCs built by NGOs are damaged by conflict or flooding; widespread lack of water, sanitation and electricity.
Human resources	Severe shortage of trained staff: very few doctors and midwives; heavy reliance on CHWs and nurses; urban–rural imbalance.
Supplies	Frequent stock-outs of essential drugs and vaccines; weak cold chain in particularly remote areas. High donor dependence.
Service scope	Primary care is still focused mainly on maternal and child health, malaria and infectious diseases; limited integration of mental health, non-communicable diseases (NCDs) or disability care.
Financing	Health budget ~2% – 3% of annual total government expenditure; extremely high donor dependence; national Health Pooled Fund supports most PHC services.
Quality and data	Inconsistent adherence to guidelines; limited supervision; incomplete data reporting and use for planning.

*Source:* Adapted from Yogesh R, Boulenger S, Pressman W. Southern Sudan Health System Assessment. Bethesda, MD: Health Systems 20/20 project, Abt Associates Inc; 2007

PHCUs, primary health care units; PHCCs, primary health care centres; PHC, Primary health care; NGOs, non-governmental organisations; CHWs, community health workers.

South Sudan’s primary care is the cornerstone of its fragile health system but remains under-resourced, unevenly accessible and heavily dependent on NGOs and donors. There is critical shortage of trained health workers, limited access and coverage – especially in conflict-affected and remote areas – and poor quality because of damaged infrastructure and frequent medicine stock-outs. It remains narrowly focused on maternal and child health and infectious diseases, with limited attention to mental health and chronic conditions. While there is real progress through community-based initiatives such as Boma health initiative (BHI) and donor support, sustainable primary care that can deliver universal, equitable and comprehensive services requires major investment, integration and government leadership.

## Medical education

South Sudan faces significant challenges in the medical education sector. The doctor-to-population ratio is alarmingly low, with approximately one doctor per 100 000 people. This shortage is underscored by the fact that there are only around 500 doctors with basic medical degrees, many of whom lack postgraduate training. At the time of independence in 2011, the country had 80 specialist doctors, 65 of whom were actively practicing.^12^

Even though, South Sudan has three public universities offering undergraduate medical education, postgraduate medical training remains limited. The College of Physicians and Surgeons was established in 2017. It offers Master of Medicine (MMED) in Surgery and MMED in Obstetrics and Gynaecology. However, it has been struggling to graduate its first cohort of specialists for eight years now. Recently, the Association of Obstetricians and Gynaecologists of South Sudan has been accredited by the East Central and Southern Africa Colleague of Obstetrics and Gynaecology (ECSA-COG) to provide a fellowship programme at Juba Teaching Hospital, and first cohort has completed her first year. It is worth mentioning that, in 2024, the University of Juba, School of Medicine launched first of its kind, MMED programme in Paediatrics and Child Health by the Department of Pediatrics and Child Health. Unfortunately, Department of Community Medicine still offers undergraduate programme. There are at least eight family physicians in the country who completed their specialist training from various institutions in East Africa, Sudan and beyond the region. They provide academic services at both the University of Juba and Upper Nile University in the Department of Community Medicine. This group is in the process of forming the South Sudan Association of Family Physicians (SSAFP), which will advocate for and promote Family medicine (FM) training within the country. To enhance its visibility, SSAFP looks forward to joining regional and global organisations of FM and primary care such as the World Organisation of Family Doctors (WONCA), the East, Central and Southern Africa College of Family Physicians (ECSA-CFP) and the Primary Care and Family Medicine (PRIMAFAMED) network of over 40 academic departments of FM across 25 countries in sub-Saharan Africa (SSA).

## Family medicine in sub-Saharan Africa

Family medicine is considered as a newly evolving discipline in the region. A scoping review by Flinkenflögel et al. reported that it was first established in South Africa and Nigeria, followed by Ghana, several East African countries and more recently additional Southern African countries.^13^ This review has shown that there is variability in the roles and responsibilities of family physicians (FPs) between and within countries. This depends on the requirements of the health system structure and available circumstance in the area. Thus, they can be stationed in different levels of the health system such as district hospitals where there are no specialists or act as supervisors and mentors for health facilities in a district.^13^ Furthermore, there are disparities within the public and private sectors. It is observed that PHC services are mainly provided by nurses or physician assistant relative to general practitioners in the private sector.^14^ Nevertheless, FPs are found to cover a wider range of health care services from hospitals, as clinicians, consultants to capacity builders, clinical trainers, leaders, managers and governance.^15^

Qualification for a family physician requires postgraduate training. A handful of sub-Saharan Africa countries such as Ghana, Botswana, Uganda, Kenya and Nigeria have developed and established training programmes.^15^ These countries have played an immense role in increasing experts in the continent even though the number remains relatively few. More countries have picked interest in starting the programme. However, there are hindrances to education and training for the FM programme. This includes, but not limited to, a lack of academic experts, insufficient or lack of learning environments and challenges in deployment of new graduates into the health system.^15^ In order to overcome these barriers, countries are required to scale up advocacy for FPs roles in the health system and training programme with higher education, regulatory bodies, departments of health and other health professional bodies.^15^

## Potential role of family medicine in South Sudan

In a post-conflict environment such as South Sudan, FM and PHC can enhance the country’s fragile healthcare system by improving access to care and providing comprehensive, continuous and person-centred cost-effective services. These approaches, which emphasise disease prevention, health promotion and managing common health issues at the community level, will reduce the strain on higher-level facilities. Family medicine can also address maternal and child health, support workforce development and improve service integration. By prioritising equitable care, particularly for underserved and marginalised groups, FM can help reduce health disparities. Furthermore, it can build resilience of health systems during crises by enhancing responses to disease outbreaks, public health emergencies, conducting practice-based research and advocating for better community resource management.

Opportunities for developing FM in South Sudan include:

- The healthcare system in South Sudan is well suited for FM, as over 90% of healthcare consumers need primary care.
- Public universities in South Sudan currently offer community medicine at the undergraduate level, amidst limited or non-existent postgraduate medical training. Community medicine forms a foundation for postgraduate FM training.
- The establishment of the South Sudan Association of Family Physicians (SSAFP), which is dedicated to promoting FM practice, postgraduate medical training and linking South Sudan with regional organisations such as ECSA-CFP and PRIMAFAMED.
